# Hemispheric Asymmetry for Affective Stimulus Processing in Healthy Subjects–A fMRI Study

**DOI:** 10.1371/journal.pone.0046931

**Published:** 2012-10-08

**Authors:** Esther Beraha, Jonathan Eggers, Catherine Hindi Attar, Stefan Gutwinski, Florian Schlagenhauf, Meline Stoy, Philipp Sterzer, Thorsten Kienast, Andreas Heinz, Felix Bermpohl

**Affiliations:** 1 Department of Psychiatry and Psychotherapy, Charité - Universitätsmedizin Berlin, Campus Mitte, Berlin, Germany; 2 Berlin School of Mind and Brain, Berlin, Germany; University of Groningen, The Netherlands

## Abstract

**Background:**

While hemispheric specialization of language processing is well established, lateralization of emotion processing is still under debate. Several conflicting hypotheses have been proposed, including right hemisphere hypothesis, valence asymmetry hypothesis and region-specific lateralization hypothesis. However, experimental evidence for these hypotheses remains inconclusive, partly because direct comparisons between hemispheres are scarce.

**Methods:**

The present fMRI study systematically investigated functional lateralization during affective stimulus processing in 36 healthy participants. We normalized our functional data on a symmetrical template to avoid confounding effects of anatomical asymmetries. Direct comparison of BOLD responses between hemispheres was accomplished taking two approaches: a hypothesis-driven region of interest analysis focusing on brain areas most frequently reported in earlier neuroimaging studies of emotion; and an exploratory whole volume analysis contrasting non-flipped with flipped functional data using paired t-test.

**Results:**

The region of interest analysis revealed lateralization towards the left in the medial prefrontal cortex (BA 10) during positive stimulus processing; while negative stimulus processing was lateralized towards the right in the dorsolateral prefrontal cortex (BA 9 & 46) and towards the left in the amygdala and uncus. The whole brain analysis yielded similar results and, in addition, revealed lateralization towards the right in the premotor cortex (BA 6) and the temporo-occipital junction (BA 19 & 37) during positive stimulus processing; while negative stimulus processing showed lateralization towards the right in the temporo-parietal junction (BA 37,39,42) and towards the left in the middle temporal gyrus (BA 21).

**Conclusion:**

Our data suggests region-specific functional lateralization of emotion processing. Findings show valence asymmetry for prefrontal cortical areas and left-lateralized negative stimulus processing in subcortical areas, in particular, amygdala and uncus.

## Introduction

The two hemispheres of the brain differ in structure and function. While hemispheric specialization of some cognitive domains such as language is well established [1], lateralization of emotion processing is still under debate. According to the right hemisphere hypothesis of emotion, the right hemisphere of the brain is specialized for emotional and the left for cognitive processes [2], [3]. This hypothesis is supported by findings from behavioral [2], [4] and lesion studies [5] although conflicting findings have been reported [6], [7], [8].

According to the valence asymmetry hypothesis [9], positive or approach-related emotions are lateralized towards the left and negative or withdrawal-related emotions towards the right hemisphere, especially in prefrontal brain regions [10], [11]. This hypothesis has found support from lesion [12], [13], [14], electroencephalography (EEG) [10], [15], [16], transcranial magnetic stimulation (TMS) [17], [18] and functional neuroimaging studies [19], [20]. However, a number of neuroimaging studies did not support the valence asymmetry hypothesis [21], [22], [23], especially when examining subcortical regions like the amygdala [24], [25], [26]. Lateralization of emotions may thus differ between prefrontal and subcortical areas [27].

Discrepancy between neuroimaging findings may, in parts, be related to a methodological issue [28]: The lateralization debate largely draws on studies that did not intend and were not designed to test for hemispheric asymmetry. Findings tend to be considered asymmetric when voxels in one hemisphere exceed statistical threshold while homologous voxels in the contralateral hemisphere do not. However, a direct comparison between hemispheres is necessary to demonstrate statistically significant lateralization. Only few investigations have directly compared hemispheres, mostly concentrating on the amygdala [29], [30].

Direct comparison between hemispheres has been provided by a pioneering meta-analysis on emotion processing [31]. This analysis does neither support the right hemisphere theory of emotion nor the valence asymmetry hypothesis. It rather suggests that the direction of lateralization is region-specific. Specifically, Wager et al. [31] found a trend towards left lateralization for approach-related emotions in the lateral prefrontal cortex. Furthermore, negative emotions were lateralized towards the left in the amygdala and towards the right in the basal ganglia. While meta-analyses can address research questions with greater statistical power than individual studies, they also entail certain limitations: meta-analyses combine studies that differ with regard to affective stimulus material (e.g., visual stimuli, auditory stimuli, recall), task demands, control conditions, imaging data analysis, and statistical thresholds. In addition, meta-analyses draw conclusions from the frequency of reported peaks which could, in principle, differ from the magnitude of activation considered in individual studies [31]. Finally, meta-analyses of functional imaging data cannot address the problem that investigation of functional asymmetry could potentially be affected by anatomical asymmetry.

The aim of the present study was to investigate functional lateralization by directly comparing BOLD responses to affective stimuli between hemispheres. A homogenous sample of 36 healthy participants underwent a standard affective stimulus processing paradigm for fMRI (International Affective Picture System) [32]. Two approaches were taken to study functional asymmetry: First, a region of interest analysis was carried out, focusing on brain areas most frequently reported in earlier neuroimaging studies of emotion [33]. Second, we performed an exploratory whole volume analysis, contrasting non-flipped with flipped fMRI data using a paired t-test. The present study focused on functional asymmetries. Because functional differences can interfere with anatomical differences [34], [35], we placed particular emphasis on normalizing our data on a symmetrical template to avoid confounding effects of anatomical asymmetries [36]. We tested three competing hypotheses: The right hemisphere hypothesis, the valence asymmetry hypothesis, and the region-specific lateralization hypothesis.

## Materials and Methods

### Participants

Thirty-six right-handed [37] healthy volunteers (18 women; mean age: 36.7 years; ±10.9 S.D.; range: 22–61 years) participated in this study. Participants were recruited through advertising. Participants had no psychiatric axis I or II disorder (SCID-interview). Exclusion criteria were current neurological or severe medical disorder, history of head injury resulting in loss of consciousness, age below 18 or above 65 years and contraindications to MRI. The study was approved by the research ethics committee of the Charité Campus Mitte and written informed consent was obtained from all study participants.

### Experimental Design

During fMRI participants viewed standardized photographs taken from the *International Affective Picture System (IAPS)* [32] as previously described [38], [39]. The IAPS is an established stimulus set to elicit emotional responses. Pictures were selected in a manner so that their standard valence scores were clearly positive, negative or neutral: the mean normative valence ratings were 7.4 (±0.5, S.D.), 2.6 (±0.9), and 5.0 (±0.4) for positive, negative, and neutral pictures, respectively. The mean normative arousal ratings were 5.0 (±0.8), 5.7 (±1.0), and 2.7 (±0.5) for positive, negative, and neutral pictures, respectively. Each of three picture conditions (positive, negative, and neutral) comprised 36 trials presented over 2 runs. Prior to half of the photographs, attention-directing cues were presented, indicating the emotional valence of the subsequent affective picture. These cue periods represent conditions of no interest in the present analysis. Photographs were presented for 2 s using an event-related design and were arranged in a pseudorandomized and counterbalanced order with respect to valence condition. Each photograph was followed by a blank screen baseline period (randomly jittered between 4.6 and 9.2 s). Subjects were instructed to confirm each picture by button press in order to keep participants engaged in the experiment and to monitor the attentiveness of the subjects. No judgment task was associated with the button response. After the fMRI session, the photographs were presented again and subjects rated each picture’s valence and intensity using a 9-point visual analogue scale. Valence and arousal ratings were not obtained in seven participants.

### FMRI Data Acquisition

MR images were acquired on a 1.5-Tesla scanner (Magnetom VISION Siemens®) with an Echo Planar Imaging (EPI) sequence (TE = 40 ms, TR = 2,3 s, α = 90°, matrix = 64×64, voxel size = 4×4×3,3 mm^3^). A total of 290 T2*-weighted images were acquired per run. Additionally, a 3D Magnetization Prepared Rapid Gradient Echo (TR = 9.7 ms, TE = 4 ms, flip angle 12°, matrix = 256×256, voxel size = 1×1×1 mm^3^) image data set was acquired.

### fMRI Data Analysis

#### Data preprocessing

FMRI data were analyzed using SPM 8 (Wellcome Department of Imaging Neuroscience, London). The functional images were slice time corrected and realigned to the mean functional image [40]. Each participant’s structural T1 image was coregistered to the mean functional image of the participant. Coregistered T1 images were segmented and spatially normalized to a group-specific symmetrical template. Such a symmetrical template was used to eliminate structural asymmetries which could potentially interfere with functional asymmetries [35]. To create the symmetrical template, first all anatomical images were normalized to the standard template provided by the Montreal Neurological Institute (MNI template) as implemented in SPM8. In the next step, all normalized anatomical images were copied and flipped. Then, a mean image of all flipped and non-flipped anatomical images was constructed and smoothed with a 10-mm full-width at half-maximum (FWHM) Gaussian kernel. All functional images were normalized to this symmetrical template, then copied and flipped. Non-flipped and flipped functional images were smoothed with a 15-mm full-width at half maximum Gaussian kernel in order to avoid false positive effects due to small anatomical differences between hemispheres.

#### First-level analysis

For first level analysis, flipped and non-flipped images were analyzed separately in the context of the general linear model approach, using the onset of each picture for a box-car function (2 s stimulus duration) to provide a stimulus function. The stimulus functions were convolved with the canonical hemodynamic response function as implemented in SPM 8 [41]. Picture valence (positive, negative and neutral) was modeled as the explanatory variable and realignment parameters were additionally included. After model estimation for each participant, contrasts of parameter estimates of stimulus-related responses were obtained at each voxel for each regressor [40].

Non-flipped functional images were used for the region of interest analysis. For the whole volume analysis, flipped and non-flipped functional images were compared using a paired t-test.

#### Region of interest analysis

Regions of interest (ROIs) were chosen based on the frequency of reported activations in earlier neuroimaging studies of emotion, as reviewed by Phan et al. [33]: regions that had shown activations in more than 25% of emotion studies were included as ROIs: Dorsolateral prefrontal cortex (BA 9/46), medial prefrontal cortex (BA 10), anterior cingulate cortex (BA 24/32), occipital cortex (BA 17/18/19), insula, basal ganglia (caudate nucleus, putamen, pallidum), parahippocampal gyrus, uncus, amygdala, and cerebellum. For all ROIs, we generated symmetrical templates from the union of the flipped and unflipped mask images of each ROI. Mask images were taken from the Brodman Atlas [42], [43]. If a ROI was not provided by the Brodman Atlas, we used the Lobes Atlas, Labels Atlas, or Automated Anatomical Labeling Atlas in descending priority [42], [43], [44].

The VOI tool (SPM 8) was used to extract the mean contrasts of parameter estimates (‘positive > neutral’, ‘negative > neutral’) of each ROI for each participant separately in the right and the left hemisphere. ‘Neutral picture viewing’ was chosen as control condition because it allows subtracting the general effect of picture viewing thus isolating the specific effect of positive or negative affective picture viewing. A paired t-test was applied to compare effects between hemispheres (SPSS 18). For the ROI analysis, results with *p*<0.05 were considered significant.

Given the small size and the shape of some of the ROIs (e.g., the amygdala) as well as the voxel size of 4×4×3,3 mm^3^ and the smoothing kernel of 15 mm, it is acknowledged that our ROI analysis does not allow to completely isolate activation in small ROIs from activation in adjacent structures (e.g., hippocampus and parahippocampal gyrus).

#### Whole volume analysis

For exploratory purposes, a whole volume second-level random effects analysis was carried out, comparing non-flipped with flipped functional data (paired t-test). This allowed the direct comparison of BOLD responses between hemispheres. Statistical parametric maps were estimated for the contrasts ‘positive > neutral picture viewing’, ‘negative > neutral picture viewing’, and ‘negative >positive picture viewing’, comparing right with left hemispheric functional data. Again, picture conditions were chosen as control conditions to subtract out general effects of picture viewing. For the whole-volume analysis, statistical significance threshold was set to *p*<0.001, uncorrected, voxel level with an extent threshold *k*≥ = 5 voxels [45]. We indicate where results survive corrections for multiple comparisons at *p*<0.05 (false discovery rate, FDR) [46].

To explore the impact of the control condition on lateralization effects, additional whole volume analyses were carried out contrasting positive and negative picture conditions with baseline (‘positive picture viewing > baseline’, ‘negative picture viewing > baseline’).

## Results

### Behavioural Data

Response times for button responses to pictures during fMRI were 911 ms (±377, S.D.), 936 ms (±385), and 912 ms (±357) for positive, negative, and neutral pictures, respectively. Response times showed no valence effect (*F*(2,70) = 0.49, *p* = 0.62).

The mean *posthoc* valence ratings differed significantly between positive (mean valence rating ± SD: 7.2±1.0), negative (2.7±0.8), and neutral (5.3±0.7) pictures (*F*(2,56) = 197.3, *p*<0.001). Positive pictures received significantly higher valence ratings than negative (*F*(1,28) = 261.2, *p*<0.001) and neutral (*F*(1,28) = 93.0, *p*<0.001) pictures. Valence ratings were significantly lower in negative compared to neutral pictures (*F*(1,28) = 171.6, *p*<0.001).

The mean *posthoc* arousal ratings differed significantly between positive (mean arousal rating ± SD: 4.8±1.6), negative (5.5±1.7), and neutral (2.5±1.5) pictures (*F*(2,56) = 37.7, *p*<0.001), with lower arousal ratings in neutral compared to positive (*F*(1,28) = 59.7, *p*<0.001) and to negative (*F*(1,28) = 56.5, *p*<0.001) pictures. There was a trend for higher arousal ratings in negative compared to positive pictures (*F*(1,28) = 3.4, *p*<0.077).

### ROI Analysis

**Figure 1:**
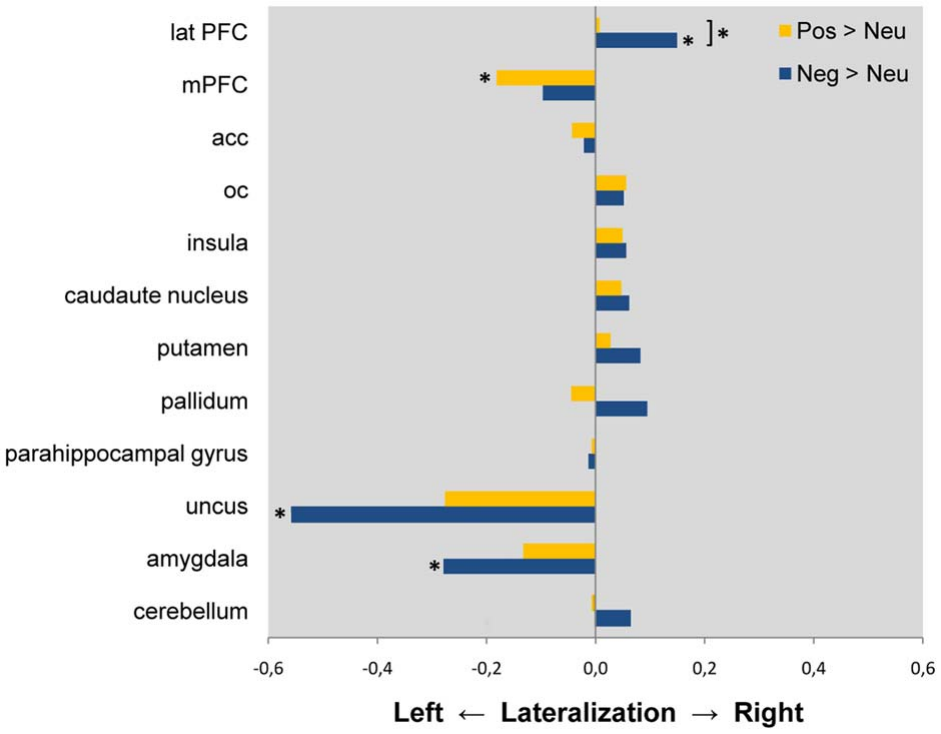
Lateralization during positive and negative picture viewing. ROI analysis. Lateralization of BOLD responses in regions of interest. The bars represent hemispheric differences of contrasts of parameter estimates (red: ‘positive > neutral’; blue: ‘negative > neutral’) extracted from the respective ROI (mean values for the respective ROI, averaged across the 36 participants). Bars extending to the left indicate lateralization towards the left (left > right), and bars extending to the right indicate lateralization towards the right (right > left). *p<0.05; (*) *p*<0.1 on tests of laterality within valence condition (positive or negative), or between positive and negative valence conditions if marked by brackets. Lat PFC, dorsolateral prefrontal cortex; mPFC, medial prefrontal cortex; acc, anterior cingulate cortex; oc, occipital cortex.

Results of the ROI analysis are shown in Figure 1. Positive (compared to neutral) stimulus processing was lateralized towards the left in the medial prefrontal cortex (BA 10; *p* = 0.006). Negative (compared to neutral) stimulus processing was lateralized towards the right in the dorsolateral prefrontal cortex (BA 9 & 46; *p* = 0.016) and towards the left in the amygdala (*p* = 0.047) and uncus (*p* = 0.013). Lateralization effects differed significantly between positive and negative stimulus processing in the dorsolateral prefrontal cortex (BA 9 & 46; *p* = 0.011), with more pronounced lateralization towards the right during negative pictures. This significant valence by laterality interaction was still present after controlling for differences in arousal between negative and positive pictures (*p* = 0.006).

### Whole Volume Analysis

**Figure 2:**
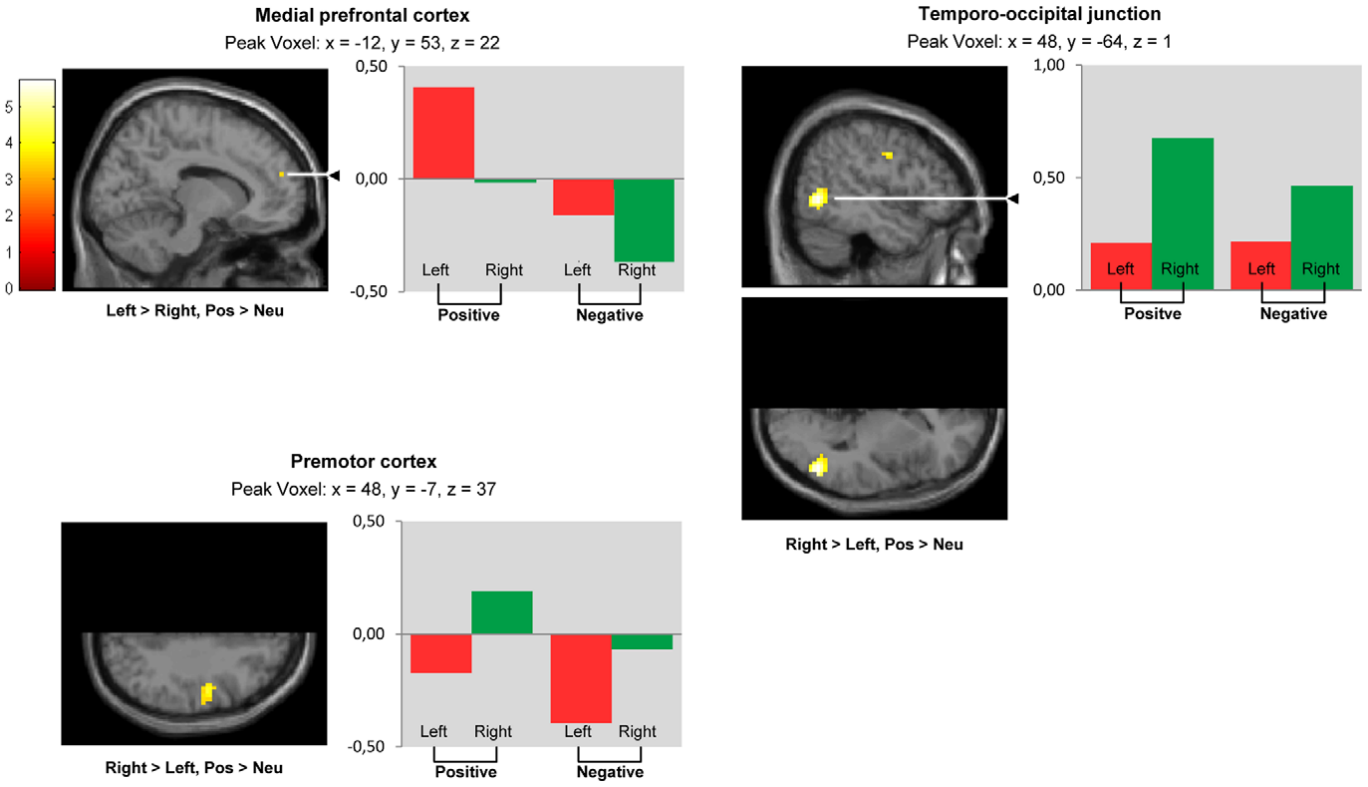
Lateralization during positive picture viewing. Whole volume analysis. Brain areas showing lateralization (‘left > right’, ‘right > left’) during positive (versus neutral) picture viewing (‘pos > neu’); SPM whole volume analysis (paired t-test) comparing non-flipped with flipped functional data. Because data represent comparisons between left and right hemispheres, sections views display half of the brain only: *‚*Left > right’ indicates clusters showing significantly larger activation in the left compared to the right hemisphere. *‚*Right > left’ indicates clusters showing significantly larger activation in the right compared to the left hemisphere. Clusters are projected on a symmetrical MNI template. *P*<0.001 uncorrected, cluster size k≥5, t = t-value. Bars represent contrasts of parameter estimates for ‘positive > neutral picture viewing’ (‘positive’) and ‘negative > neutral picture viewing’ (‘negative’). Values were extracted from peak voxels presented in Table 1 for the right and the left hemisphere averaged across the 36 participants.

For exploratory purposes, a SPM whole volume analysis was carried out to directly compare BOLD responses between the right and the left hemisphere. A paired t-test was used to compare non-flipped versus flipped functional images that were normalized on a symmetrical template. Comparing ‘positive > neutral pictures’ (Figure 2, Table 1), we observed lateralization towards the left in the medial prefrontal cortex (BA 9) and towards the right in the premotor cortex (BA 6) and the temporo-occipital junction (BA 19 & 37).

**Table 1:** Contrast ‘positive > neutral picture viewing’.

Anatomical Region	Hemisphere	Coordinates (MNI)			Max T Value	Cluster Size (k)
		X	Y	Z		
Temporo-occipital junction (inferior & middle temporal gyrus; middle occipital gyrus; BA 19, 37)*	Right > Left	48	−64	1	5.70	91
Premotor cortex (precentral gyrus, BA 6)	Right > Left	48	−7	37	4.17	24
Medial prefrontal cortex (superior & medial frontal gyrus; BA 9)	Left > Right	−12	53	22	3.51	5

Maximum t-values and peak voxel coordinates for activation clusters, uncorrected *p*<0.001, k≥5; * activations survive FDR-correction at *p*<0.05. Within each row, anatomical regions and BAs were sorted according to their proportion in the respective cluster (descending order).

BA = Brodmann Area, k = number of voxels.

**Figure 3:**
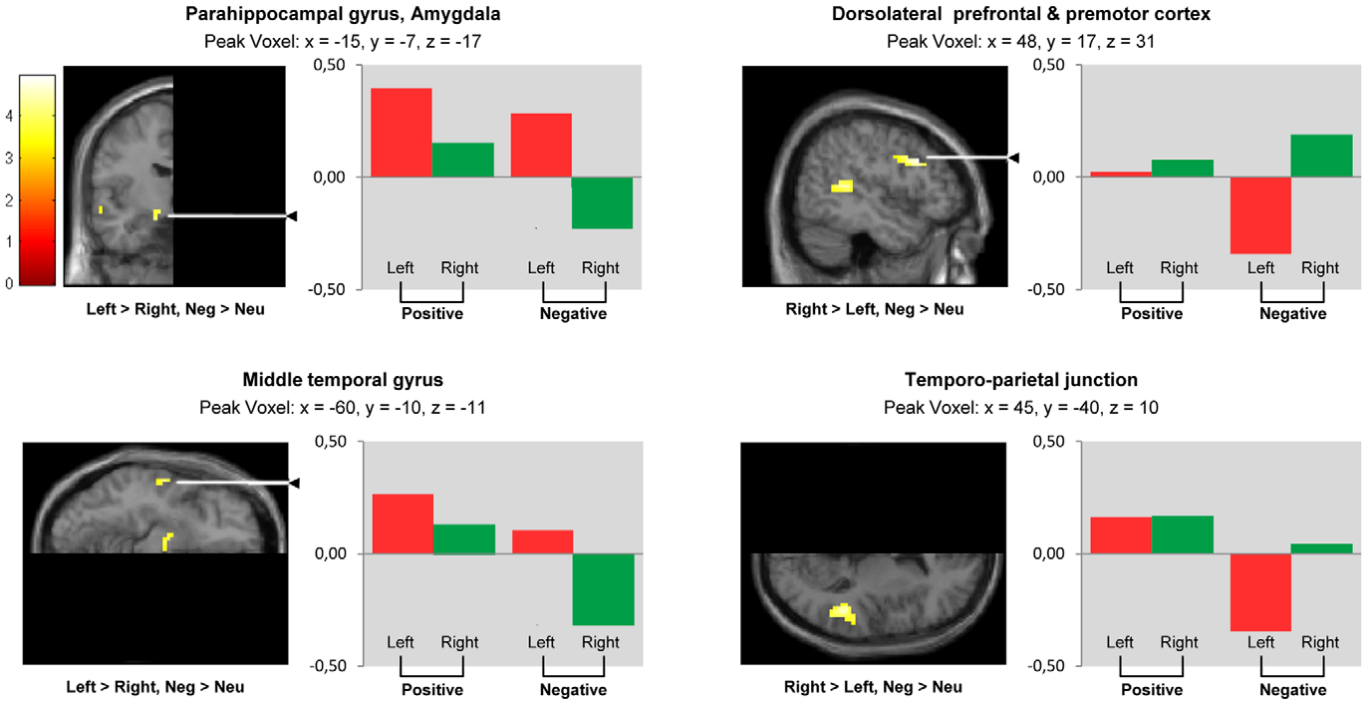
Lateralization during negative picture viewing. Whole volume analysis. Brain areas showing lateralization (‘left > right’, ‘right > left’) during negative (versus neutral) picture viewing (‘pos > neu’); SPM whole volume analysis (paired t-test) comparing non-flipped with flipped functional data. Because data represent comparisons between left and right hemispheres, sections views display half of the brain only: *‚*Left > right’ indicates clusters showing significantly larger activation in the left compared to the right hemisphere. *‚*Right > left’ indicates clusters showing significantly larger activation in the right compared to the left hemisphere. Clusters are projected on a symmetrical MNI template. *P*<0.001 uncorrected, cluster size k≥5, t = t-value. Bars represent contrasts of parameter estimates for ‘positive > neutral picture viewing’ (‘positive’) and ‘negative > neutral picture viewing’ (‘negative’). Values were extracted from peak voxels presented in Table 2 for the right and the left hemisphere averaged across the 36 participants.

In the comparison ‘negative > neutral pictures’ (Figure 3, Table 2), lateralization towards the right was found in the dorsolateral prefrontal and premotor cortex (BA 9, 46, 6) and the temporo-parietal junction (BA 37, 39, 42). Stronger left-hemispheric activations were observed in the amygdala and the paraphippocampal gyrus as well as the middle temporal gyrus (BA 21).

**Table 2:** Contrast ‘negative > neutral picture viewing’.

Anatomical Region	Hemisphere	Coordinates (MNI)			Max T Value	Cluster Size (k)
		X	Y	Z		
Dorsolateral prefrontal & premotor cortex (inferior & middlefrontal gyrus, precentral gyrus; BA 9, 46, 6)	Right > Left	48	17	31	4.93	103
Temporo-parietal junction (posterior superior & middletemporal gyrus, angular gyrus; BA 37, 39, 42)	Right > Left	45	−40	10	4.44	55
Middle temporal gyrus (BA 21)	Left > Right	−60	−10	−11	4.10	11
Amygdala, parahippocampal gyrus	Left > Right	−15	−7	−17	4.06	11

Maximum t-values and peak voxel coordinates for activation clusters, uncorrected *p*<0.001, k ≥5. Within each row, anatomical regions and BAs were sorted according to their proportion in the respective cluster (descending order).

BA = Brodmann Area, k = number of voxels.

**Figure 4:**
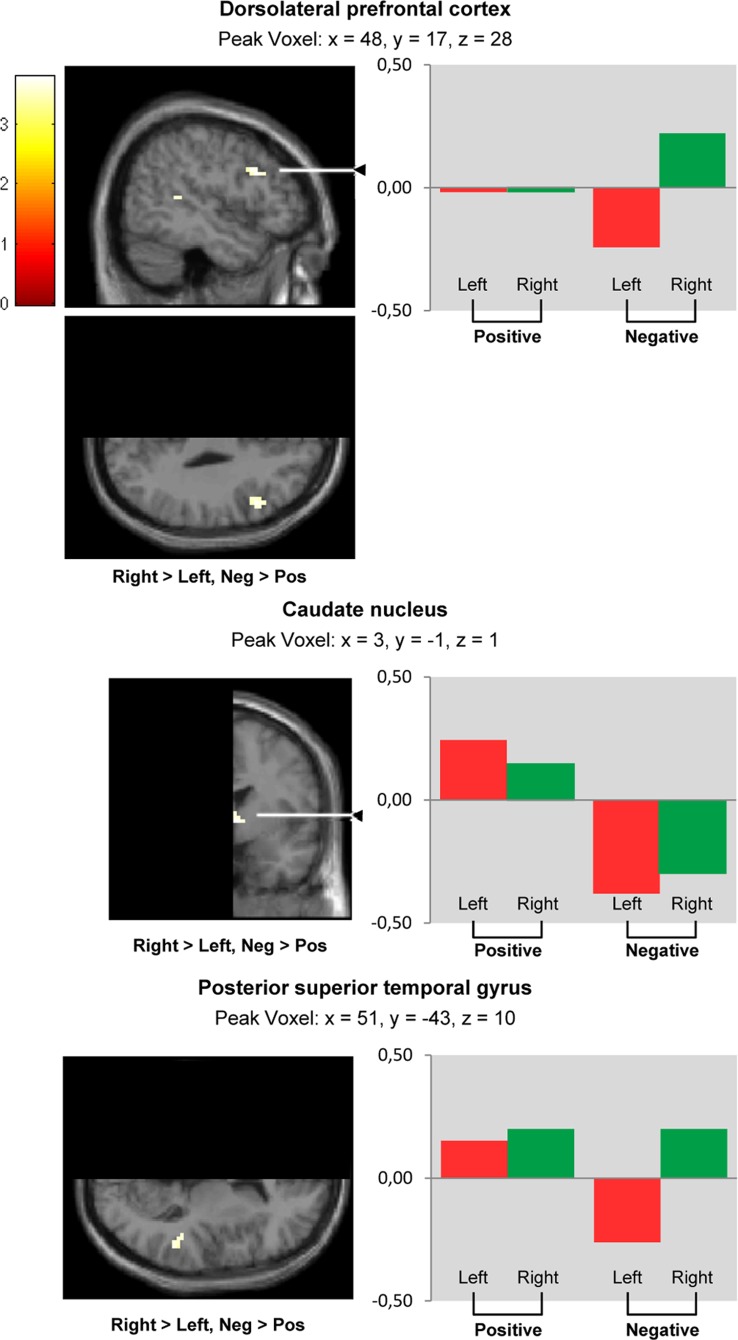
Lateralization during negative versus positive picture viewing. Whole volume analysis. Brain areas showing lateralization (‘right > left’) during negative versus positive picture viewing (‘neg >pos’); SPM whole volume analysis (paired t-test) comparing non-flipped (right-sided) with flipped (left-sided) functional data. Because data represent comparisons between left and right hemispheres, sections views display half of the brain only: *‚*Right > left’ indicates clusters showing significantly larger activation in the right compared to the left hemisphere. The reverse contrast (‘left > right’) revealed no significant effect. Clusters are projected on a symmetrical MNI template. *P*<0.001 uncorrected, cluster size k≥5, t = t-value. Bars represent contrasts of parameter estimates for ‘positive > neutral picture viewing’ (‘positive’) and ‘negative > neutral picture viewing’ (‘negative’). Values were extracted from peak voxels presented in Table 3 for the right and the left hemisphere averaged across the 36 participants.

The comparison ‘negative >positive pictures’ (Figure 4, Table 3) revealed a lateralization towards the right in the dorsolateral prefrontal cortex (BA 9, 46), the posterior superior temporal gyrus (BA 41) and the caudate nucleus.

**Table 3:** Contrast ‘negative >positive picture viewing’.

Anatomical Region	Hemisphere	Coordinates (MNI)			Max T Value	Cluster Size (k)
		X	Y	Z		
Caudate nucleus	Right > Left	3	−1	1	3.80	12
Dorsolateral prefrontal cortex (middle & inferiorfrontal gyrus; BA 9, 46)	Right > Left	48	17	28	3.72	20
Posterior superior temporal gyrus (BA 41)	Right > Left	51	−43	10	3.60	9

Maximum t-values and peak voxel coordinates for activation clusters, uncorrected *p*<0.001, k≥5. Within each row, anatomical regions and BAs were sorted according to their proportion in the respective cluster (descending order).

BA = Brodmann Area, k = number of voxels.

### Exploratory Baseline Comparisons

To explore how the control condition chosen impacts on lateralization of emotional picture processing, we performed an additional analysis using the baseline (instead of the neutral picture condition) as a control. In these baseline contrasts, both positive and negative picture viewing were generally associated with lateralization towards the right hemisphere. The contrast ‘positive picture viewing > baseline’ revealed lateralization towards the right in the lateral prefrontal cortex (BA 45, 46), the occipital lobe (BA 18, 19) and the lateral parietal lobe (BA 7, 39) extending to the temporo-parietal junction (BA 37, 39); and lateralization towards the left in the postcentral gyrus (BA 2, 3). The contrast ‘negative picture viewing > baseline’ showed lateralization towards the right in the dorsolateral prefrontal cortex (BA 9, 46), the occipital lobe (BA 19) and the lateral parietal lobe extending to the temporo-parietal junction (BA 7, 39, 40, 21, 22); and lateralization towards the left in the orbitofrontal cortex (BA 11, 47).

## Discussion

The present fMRI study provides evidence for hemispheric asymmetry of affective stimulus processing in healthy participants. However, our present data do not generally associate positive and negative emotions with one or the other hemisphere. Instead, the pattern of lateralization differs between brain regions. Specifically, positive stimulus processing is lateralized towards the left in the medial prefrontal cortex; and towards the right in the premotor cortex and temporo-occipital junction. Negative stimulus processing shows lateralization towards the left in the amygdala, uncus and middle temporal gyrus; and lateralization towards the right in the dorsolateral prefrontal cortex (extending to the premotor cortex) and temporo-parietal junction.

The present study adds to the literature in that it places particular emphasis on several issues relevant for the investigation of functional lateralization. First, we directly compare BOLD responses measured in one hemisphere with the corresponding responses in the other hemisphere. Such direct comparison is necessary to demonstrate statistically significant lateralization and is preferable to an approach that argues for asymmetry when voxels in one hemisphere exceed statistical threshold while homologous voxels in the opposite hemisphere do not [28]. Second, we perform ROI analyses motivated by a priori hypotheses as well as more exploratory whole-volume analyses. This allows systematic and comprehensive investigation of lateralization. Most previous studies providing direct comparisons between hemispheres have concentrated on the amygdala [29]. Third, we rule out that functional asymmetries are confounded by structural asymmetries. Because structural differences between hemispheres can give a false impression of or obscure functional differences [34], [35], [36], we normalize our functional data on a group-specific symmetrical template.

### Valence Asymmetry Hypothesis

The valence asymmetry hypothesis of emotion posits that the left hemisphere is dominant for positive and the right for negative emotions. The hypothesis has found particular support from lesion, EEG, TMS and functional neuroimaging findings in the prefrontal cortex [10], [12], [13], [14], [15], [16], [20], [21], [22], [23]. However, subcortical findings have not consistently supported the hypothesis [24], [25], [26]. In accordance with this, we found support for valence asymmetry in the prefrontal cortex, but not in subcortical brain areas (e.g., amygdala, uncus).

Extending earlier prefrontal findings, our data suggest that lateralization of emotion processing might differ between the medial (BA 10) and the dorsolateral prefrontal (BA 9, 46) cortex: Positive stimuli induce left-lateralization in the medial prefrontal cortex, but no significant lateralization in the dorsolateral prefrontal cortex. In contrast, negative stimuli provoke right-lateralization in the dorsolateral prefrontal cortex, but no significant lateralization in the medial prefrontal cortex.

Not consistent with the valence asymmetry hypothesis (in the above version), the amygdala and uncus show left-lateralization during negative stimulus processing, rather compatible with earlier suggestions that negative emotions are lateralized towards the left in limbic brain areas [47].

### Role of the Control Condition

Our main analyses used neutral picture viewing as a control for positive and negative picture conditions. Strikingly different findings were obtained when we used the blank screen baseline condition as a control in the whole volume analysis. Specifically, both positive and negative picture viewing showed lateralization towards the right in several cortical areas (including the lateral prefrontal cortex, the temporo-parietal junction and the occipital lobe) when contrasted with the blank screen baseline. At first glance, these findings seem to support the right hemisphere theory of emotion [2], [3]. However, since picture and, in particular, face processing is lateralized towards the right [48], [49], it is more likely that the right lateralization observed in these baseline contrasts is driven by picture rather than by emotion processing. Our finding illustrates how lateralization effects may vary depending on the control condition chosen, particularly in studies presenting pictorial stimulus material.

### Lateralization in the Amygdala

Due to its important role in emotion processing, several earlier fMRI studies have searched for lateralization effects in the amygdala. Findings have been inconsistent, even in studies directly testing for condition-by-hemisphere interaction: Some studies report lateralization towards the left [29], [50], others towards the right [51] and still others bilateral activation [52], [53] during negative stimulus processing. These discrepant findings are possibly due to differences in fMRI paradigms: Amygdala activation seems to be lateralized towards the left when visible (non-masked) negative pictures or language-related affective stimuli are presented, while invisible (masked) affective stimuli seem to produce greater right-sided activation [31], [54], [55], [56], [57]. It has therefore been proposed that the right amygdala is associated with rapid stimulus detection and the left amygdala with more detailed affective information processing [58]. Thus, the relatively long presentation of (non-masked, visible) stimuli might account for the left-lateralized amygdala responses to negative pictures observed in the present study. With regard to positive stimulus processing, our findings in both ROI (Figure 1) and whole brain analysis (Figure 3) are compatible with left-lateralized amygdala responses also to positive pictures, although this effect appears to be weaker (compared to the negative picture condition) and did not reach significance here.

### Conclusion and Perspective

The present study investigated functional lateralization of emotion processing in healthy subjects. Methodologically, particular emphasis was placed on the direct comparison between left and right hemispheric functional data, the systematic and comprehensive investigation of relevant brain areas (through both hypothesis-driven ROI and exploratory whole brain analysis), and the normalization of functional data to a symmetrical template. This approach revealed region-specific lateralization during passive IAPS picture viewing. Specifically, our data suggest valence asymmetry in prefrontal cortical areas and left-lateralized negative stimulus processing in subcortical brain areas, in particular amygdala and uncus. However, the pattern of lateralization observed here during passive IAPS picture viewing may not generalize to all forms of emotion processing. The approach taken here to study lateralization could be useful for further investigation of factors that influence lateralization of emotion processing. Such factors might include stimulus material (e.g., visual, auditory, olfactory), stimulus duration and level of awareness (e.g., visible, invisible). Lateralization may also be influenced by induction method (e.g., perception versus imagery), cognitive demand (e.g., passive viewing versus judgment task), and social content (e.g., emotional stimuli with versus without social information). In addition, lateralization might differ between approach- and withdrawal-related emotions (as opposed to positive and negative) [59] or between individual emotions (e.g., happiness, anger, disgust). Finally, the approach taken here to study lateralization could shed new light on the yet controversial hemispheric imbalance hypothesis of depression [60] which postulates hypoactivity in the left relative to the right prefrontal cortex during emotion processing in depression.
